# Editorial: Insights in functional and applied plant genomics: 2023

**DOI:** 10.3389/fpls.2025.1615289

**Published:** 2025-05-08

**Authors:** Huihui Li

**Affiliations:** ^1^ State Key Laboratory of Crop Gene Resources and Breeding, Institute of Crop Sciences, Chinese Academy of Agricultural Sciences (CAAS), CIMMYT‐China Office, Beijing, China; ^2^ Nanfan Research Institute, Chinese Academy of Agricultural Sciences (CAAS), Sanya, Hainan, China

**Keywords:** plant genomics, transcription factors (TFs), genome assembly, genomic prediction, deep learning

Recent advances in plant genomics have profoundly deepened our understanding of plant biology and accelerated efforts to address key agricultural challenges. The increasing availability of high-quality reference genome sequences for nearly all major crop species (Xie et al., 2024), alongside the widespread adoption of multi-omics platforms and cutting-edge gene-editing technologies (Rönspies et al., 2021), is enabling unprecedented insights into gene functions underlying critical phenotypic traits. These developments are paving the way for innovative strategies in crop improvement. However, the full potential of these datasets remains constrained by limitations in computational tools—particularly the lack of efficient algorithms for extracting biologically meaningful insights from large-scale datasets and the scarcity of robust machine learning models for accurate phenotype prediction in genomic selection (Farooq et al., 2024). Addressing these challenges will require parallel advances in biological and bioinformatics research to further accelerate the genetic improvement of crop plants.

Over the past decade, the field of crop genomics has made remarkable strides. As one of the most dynamic areas within plant sciences, it holds immense promise for ensuring food security and advancing sustainable agricultural development. This Research Topic, *Insights in Functional and Applied Plant Genomics: 2023*, was dedicated to exploring novel insights, emerging methodologies, ongoing challenges, and future directions in functional and applied plant genomics. The Research Topic features nine manuscripts, including seven original research articles, one review, and one systematic review. These contributions collectively span a wide range of plant systems, covering recent discoveries in both major crops like wheat (*Triticum aestivum* L.) and underrepresented species such as fig, rose, *Lindenbergia philippensis*, mungbean, and alfalfa.

A central theme in this Research Topic is the role of transcription factors (TFs) in regulating gene expression and phenotypic traits. Several articles explore the multifaceted functions of TFs across diverse plant species. For instance, Zheng et al. provide a comprehensive review of the GOLDEN2-LIKE (GLK) TFs, which are functionally redundant nuclear regulators in the GARP subfamily of MYB transcription factors. GLKs are known to govern genes involved in photosynthesis and chloroplast biogenesis. Previous studies have shown that GLK knockout mutants display abnormal chloroplast structures without a complete loss of chloroplast formation. Zheng et al. synthesized current knowledge on the pleiotropic roles of GLKs, underscoring their broader functional relevance in plant biology.

Similarly, WRKY transcription factors—another major family—are explored for their key roles in plant development, stress responses, and secondary metabolite biosynthesis. In the medicinal plant *Erigeron breviscapus*, a species valued for its flavonoid content and therapeutic use in cardiovascular and cerebrovascular treatments, Song et al. identified 75 WRKY TFs through genome-wide analysis. Notably, 74 of these responded to exogenous treatments with abscisic acid and salicylic acid, and several were upregulated following gibberellin 3 (GA₃) application. Functional analysis revealed that many of these TFs were involved in flavonoid biosynthesis pathways. This study advances our understanding of WRKY-mediated regulation of secondary metabolism and offers promising avenues for breeding E. breviscapus cultivars with enhanced scutellarin content.

Alfalfa (*Medicago sativa* L.), one of the most widely cultivated forage crops, is renowned for its high tolerance to soil salinity. Li et al. conducted a genome-wide analysis of the *Zhongmu 1* cultivar and identified 114 members of the NAC transcription factor family, classifying them into 13 subgroups. Among these, subfamily V was found to play a potential role in salinity stress responses. Expression profiling revealed that *MsNAC40* plays a critical role in modulating salt stress. Functional validation through overexpression lines demonstrated significantly increased plant height, net photosynthetic rate, stomatal conductance, K^+^/Na^+^ ratio, and transpiration rate compared to controls, while leaf conductivity was significantly reduced.

WOX transcription factors, a plant-specific family, are also crucial in regulating development and stress responses. Duan et al. performed a comprehensive identification of 381 *WOX* genes in rose (*Rosa hybrida*), which were mapped across seven chromosomes. Transcriptome analysis revealed nine *RhWOX* genes with differential expression during root development, with three positively correlated with adventitious root formation. Notably, *RhWOX331* was shown to promote adventitious root primordium initiation. Overexpression of *RhWOX331* in *Arabidopsis thaliana* mitigated root growth inhibition by high concentrations of IBA and NPA, increased the number of lateral roots along the primary root, and enhanced overall plant height.

The assembly of new genome sequences from underutilized plant species provides critical insights into plant evolution and serves as a foundation for modern breeding approaches. Chen et al. assembled the genome of *L. philippensis*, an ornamental species collected from the Xishuangbanna Tropical Botanical Garden, Chinese Academy of Sciences. The assembled genome was 407.46 Mb in size, with a level of completeness comparable to 15 other species within the Lamiales order, which comprises over 23,755 species across 24 families. This assembly contributes valuable information for understanding species diversification and genome evolution in Lamiales.

Another notable genome assembly was presented by Bao et al. for fig (*Ficus carica* L.), a nutritionally important horticultural crop. Cultivated for over 11,000 years in Southwest Asia and the Middle East, figs are known for their resilience to poor soils and harsh environmental conditions. The genome, spanning 366.34 Mb and assembled into 13 chromosomes, achieved a contig N50 length of 9.78 Mb. Comparative genomic analysis revealed that *F. carica* diverged from *F. microcarpa* approximately 2–3 million years ago, likely following a whole-genome duplication event. Additionally, allelic variation in the *CHS* gene in *F. carica* was shown to influence anthocyanin biosynthesis, contributing to differences in fruit color.

Advancements in genomic prediction rely heavily on the development and refinement of machine learning (ML) models. Montesinos-López et al. evaluated the performance of a deep learning (DL) model against the widely used genomic best linear unbiased prediction (GBLUP) method using a large wheat dataset. The DL model consistently outperformed GBLUP in predictive accuracy for two traits across a five-fold cross-validation framework. In a related study, Montesinos-López et al. demonstrated that integrating environmental covariates into genomic prediction models significantly enhances accuracy by reducing prediction errors. They validated this approach in maize and rice, emphasizing the importance of incorporating environmental data in genomic prediction. Nonetheless, further research is needed to establish robust feature engineering frameworks for effectively integrating environmental variables.

Mungbean (*Vigna radiata* L.), an important legume crop widely cultivated in South Asia and arid regions of southern Europe, has gained attention for its adaptability and nutritional value. Ahmed et al. provided an in-depth review of genome-wide association studies (GWAS) in mungbean, emphasizing their role in uncovering the genetic basis of agronomic traits and enhancing crop productivity through molecular breeding.

In summary, this Research Topic highlights pivotal advances in functional and applied plant genomics, with particular emphasis on underrepresented and neglected crop species. The Research Topic offers valuable insights into genetic mechanisms, novel genome assemblies, and emerging computational approaches, underscoring their collective potential to drive future crop improvement efforts.
